# Single-Incision Cholecystectomy in about 200 Patients

**DOI:** 10.1155/2011/915735

**Published:** 2011-07-02

**Authors:** Roland Raakow, Dietmar A. Jacob

**Affiliations:** ^1^Department of Surgery, Vivantes Hospital Am Urban, Dieffenbachstrasse 1, 10967 Berlin, Germany; ^2^Department of Surgery, Vivantes Hospital Spandau, 13585 Berlin, Germany; ^3^Department of Surgery, Vivantes Hospital Spandau, Center for Minimally Invasive Surgery, Neue Bergstraße 6, 13585 Berlin, Germany

## Abstract

*Background and Aims*. We describe our experience of performing transumbilical single-incision laparoendoscopic cholecystectomy as standard procedure for acute and chronic gallbladder diseases. *Methods*. Between September 2008 and March 2010, 220 patients underwent laparoscopic single-incision surgery. A single port was used for 196 patients and two conventional 5 mm and one 10 mm port in 24 cases. All operations were performed with straight instruments. *Results*. Single-incision surgery was successfully performed in 215 patients (98%). Three patients (1.4%) required conversion to a three-port technique and two patients (0.9%) to an open procedure. Average age of 142 women (65%) and 78 men (35%) was 47 years (range: 15–89), average ASA status 2 (range: 1–3) and BMI 28 (range: 15–49). Mean operative time was 62 minutes (range: 26–174) and 57 patients (26%) had histopathological signs of acute cholecystitis. Eleven patients (5%) developed to surgery-related complications and nine (4%) of these required a reoperation. The mean followup was 331.5 (range: 11–590) days. *Conclusion*. Transumbilical single-incision cholecystectomy is a feasible and safe new approach for routine cholecystectomy. After a short learning curve, operation time and complication rate are comparable with standard multiport operation. In addition, most cases of acute cholecystitis can be performed with this technique.

## 1. Introduction

Laparoscopic cholecystectomy with three or four ports is the standard operation for gallbladder diseases worldwide. The established use of 5 or 10 mm instruments and ports leads to small skin incisions in the upper abdominal wall. However, these scars are still visible and might be a potential risk factor for incisional hernia or adhesions. Furthermore, new innovative methods such as NOTES (natural orifice transluminal endoscopic surgery) show promising first results regarding the technical feasibility and the possibility of scarless and painless operation [1–4]. At the present time, it becomes more important for younger patients to undergo surgery with none or at least very small scars. NOTES surgery might be the solution but is in case of transvaginal access to the abdominal cavity limited to female patients, and the procedure is not as easy to learn. In addition, this technique requires special instruments, which do not exist in a regular department of surgery. But due to the discussion about NOTES, another approach for the treatment of both genders is getting more attention from the public. The transumbilical access, described in the literature amongst others as laparoscopic single-site surgery (LESS) [5, 6]. For this technique, a 15 to 20 mm incision is made direct through the umbilicus, which is defined as a natural embryonic scare, and, therefore, the procedure is also called e-NOTES (embryonic natural orifice transumbilical endoscopic surgery). Beside the positive cosmetic effect of transumbilical incision, less incisional pain has been reported [7, 8]. This report describes our experience with single-incision cholecystectomies in 220 patients as standard procedure using a commercial available single-incision and conventional straight instruments. As far as we know, this is the largest series about single-incision surgery as standard procedure. 

## 2. Materials and Methods

### 2.1. Patients

Between September 2008 and March 2010, 220 laparoscopic single-incision cholecystectomies were performed at the “Vivantes Klinikum Am Urban”, Berlin, Germany by three experienced surgeons in standard technique. Single-incision operation using two 5 mm and one 10 mm trocar was performed in 24 patients (11%) between October 2008 and January 2009. Patients with symptomatic acute and chronic gallbladder disease were included in the observation, and all operations were performed consecutively. Exclusion criteria for single-incision surgery were patients with gallbladder perforation, diagnosed in ultrasound or CT scan, with peritonitis or severe critical illness. All patients were hospitalized for at least two days and were completely informed about the single-incision technique. All patients had the choice to undergo traditional three-port procedure.

### 2.2. Surgical Technique (Single Port)

**Figure 1:**
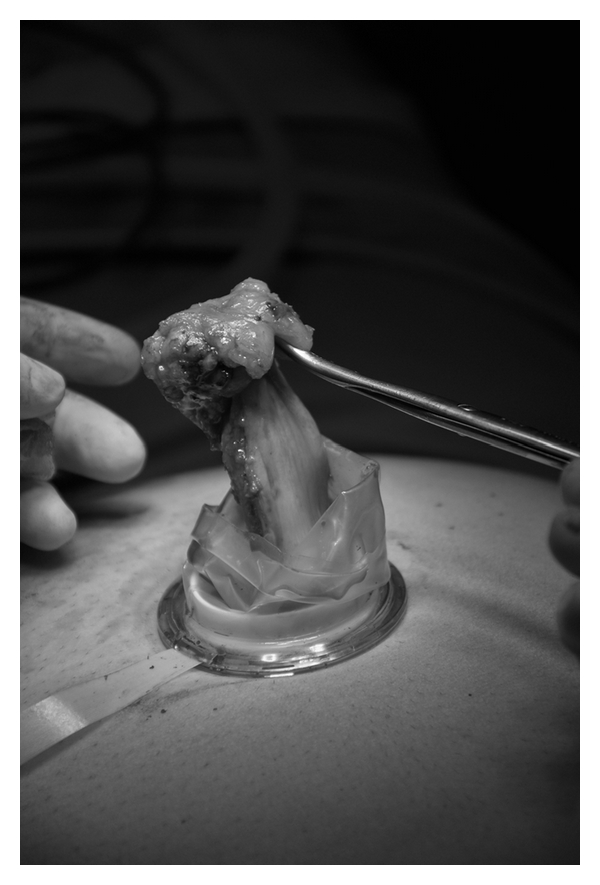
Safe removal of the gallbladder through the TriPort system without an endobag.

**Figure 2:**
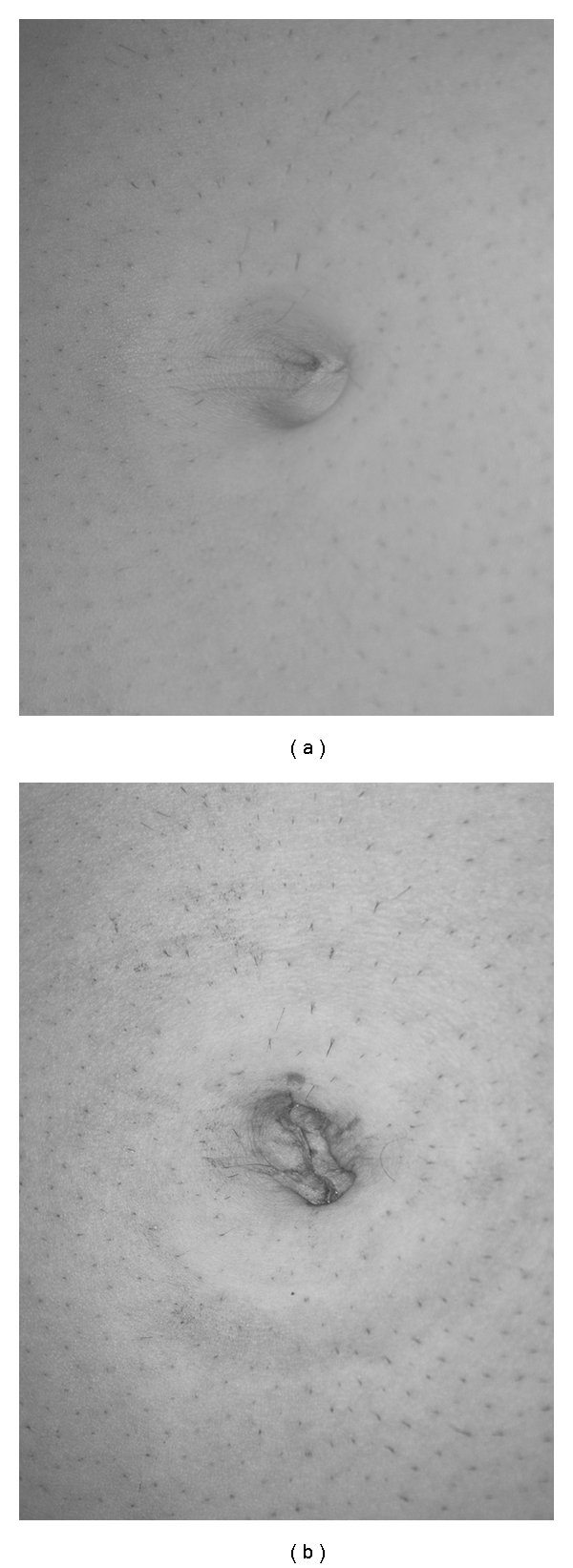
Umbilicus before (a) and after (b) single-port operation via transumbilical incision.

Operating and assisting surgeon are standing both on the left side of the patient. Both arms are rolled out and the monitor is placed on the opposite site of the surgeons near the right shoulder. The operation begins with a longitudinal incision direct through the umbilicus between both umbilical edges (approximately 1.5 to 2 cm). For a clear and safe closure of the linea alba at the end of the operation, the umbilicus has to be disconnected from the ground with a scissor. After good exposure of the linea alba with small hooks, the fascia has to cut with a scissor at a length of 15 to 20 mm. After dissolving of eventually existing adhesions with fingers, a Langenbeck`s hook has to retract the inferior part of the incision and the single-port (TriPort, Olympus, Germany) can be safely brought into the abdominal cavity with the TriPort injector introducer. After establishing the pneumoperitoneum up to 14 mm Hg with CO^2^, a 30° 5 or 10 mm laparoscope was used for initial inspection of the abdominal cavity at a 15–20° reverse Trendelenburg, right-side-up position. We used as instruments conventional straight 5 mm graspers and a 5 mm hook electrocautery device. Because of the gel valves, a continuous grease of the instruments (Instellagel, Farco-Pharma GMBH, Germany) is important for a safe, nonfitful handling. A special technique for a better movement inside the abdominal cavity is cross-handedness of the instruments. That means to undercross the instrument in the left hand under the instrument in the right hand. For a right hander, the operation will be performed with the left hand. The right hand holds the gallbladder with a grasper, and the hilum was prepared with a hook electrocautery device to expose the cystic duct and cystic artery. Both structures were clipped with a 5 mm endoscopic clip applier (Ligamax^5^ M/L, Ethicon Endo-surgery, OH, USA) and divided with scissors. After dissection the gallbladder from the fossa, the bladder was removed through the TriPort system without an endobag (Figure 1). The fascial incision was closed with a nonabsorbable 0 suture (Prolene, Ethicon, Germany). Finally, skin closure was done with an absorbable 4/0 suture (Monocryl, Ethicon, Germany). Comparison of the umbilicus before (Figure 2(a)) transumbilical incision and at the end of the operation (Figure 2(b)) demonstrates that the incision is only at the ground of the umbilicus. 

## 3. Results

### 3.1. Demographic Data

The mean age of 142 females (65%) and 78 males (35%) was 47 years (range: 15–89), and an elective surgery was planned for 154 patients (70%). The average American Society of Anesthesiologists (ASA) classification was 2 (range: 1–3) and the body mass index (BMI) 28 (range: 15–49). Mean hospital stay was 4 days (range: 2–20) and 103 patients (47%) had secondary diagnoses. Thirty-seven patients (17%) presented preoperative bile duct stones and of all received an endoscopic retrograde cholangiography (ERC). Twenty-three patients (10%) had clinical signs of biliary pancreatitis in medical history. Preoperatively measured laboratory values were in a regular range for leucocytes, C-reactive protein (CRP), aspartate aminotransferase (AST), alkaline phosphatase (AP), and bilirubin.

### 3.2. Operative Details

All patients received the same prophylactic antibiotic treatment of 2 g Cefotaxim and 0.5 g Metronidazol as single-shot dosage. Mean operation time was 62 (range: 26–174) minutes, and 31 patients (14%) had an incidential umbilical hernia. Twenty-two patients (10%) had previously undergone abdominal operation, and 54 patients (24%) showed peritoneal adhesions. Three patients (2%) required conversion to a three-port technique because of a very large gallbladder with deep positioned hilus. An exact identification of the structures in the hilum via single port was not possible. An open procedure was performed in two patients (1%). One patient had a severe bleeding out of the cystic artery after the first clip was accidentally removed. The bleeding source could not be satisfied or identified in laparoscopic technique. A second patient showed a preoperative not known cholecystoduodenal fistula. This operation could not been performed in laparoscopic technique. A wound drainage was positioned at the end of operation in five cases (3%).

### 3.3. Postoperative Complications

Eleven patients (5%) developed complications related to surgery. Seven of these patients (3%) underwent a second operation. Two patients developed a hematoma and two a seroma at the umbilicus. Their discomforts healed up without any intervention. Another two patients underwent surgery due to small incisional hernia at the umbilicus. Both hernias could be treated without a mesh. Because of wound infection, a wound debridement was undertaken in three patients. The fascia was in both cases intact. A severe complication regarding the bile duct was noticed in two patients. One patient developed a 2 cm necrosis of the bile duct after Mirizzi's syndrome, two days after the initial operation. A second patient had a bile duct leakage because of a thermocoagulation injury. A bilidigestive anastomosis was performed in both patients. The following hospital stay was prolonged but without more complications.

### 3.4. Pathology

All specimens were analyzed by two experienced pathologists in our institution. Of 220 patients, 202 (92%) had gallbladder stones and 150 patients (68%) multiple stones. Signs of gallbladder inflammation was diagnosed in 218 patients (99%), and 57 (26%) patients had an acute cholecystitis. The classification of inflammation grade was in 74 patients (34%) light, 101 patients (46%) moderate and 45 patients (20%) severe. A gallbladder hydrops was noted in 53 patients (24%), and ten patients (4.5%) developed a shrunked gallbladder. Only one patient (1%) revealed a gallbladder perforation with local peritonitis.

## 4. Discussion

We could demonstrate in one of the largest series that single-incision cholecystectomy is feasible and safe as standard technique for elective and acute gallbladder disease. Our results are on a par with conventional technique using three or four ports. Most patients in our collective were satisfied with an almost scarless procedure and less pain after operation. Previously published studies about multiport technique which included more than 1000 patients showed similar results compared to our study group [9–11]. The conversion rate to an open procedure was in former studies between 2% and 7% and in our population only 1%. Major complications in multiport surgery such as bile duct or vessel injury were noted in 0.9% to 5.8% of all patients. Although we had a comparative high complication rate of 5%, major complications like bile duct injury and necrosis happened only in two patients (1%), which is within the international standard. While single-incision surgery is getting more and more popular, patient numbers of previously published reports are still low [12–19]. A review of eight studies from 2009 about single-incision cholecystectomy shows only two studies with 100 patients [14, 17], and the largest series of single-incision surgery, published by Lee et al., included only 37 patients [12]. One of the most important points in the discussion about NOTES or single-incision surgery is the extended operation time, because of a more complicated access to the abdominal cavity and the difficult handling of the instruments. The mean operation time in eight studies about LESS surgery including 365 patients was 80 minutes (range: 51–94) [12–19]. But it is mentionable that studies with a higher case number like Rivas et al. and Hernandez et al. had a reduced operation time. One reason might be that the learning curve for this technique is certainly longer as for the three- or four-port technique. Rivas et al. compared in their study including 100 patients the first 50 patients with the second 50 ones. Age and BMI were almost equal, but the operation time was considerable reduced from 73 to 45 minutes. Our mean operation time was 64 minutes, and it is still close to the regular time for conventional laparoscopic cholecystectomy. However, we had a high percentage of patients with acute cholecystitis compared to other studies with 5% and 9% [14, 15]. A partition of our 220 cases into patients with acute and chronic cholecystitis showed a considerably different operation time. Fifty-seven patients with acute cholecystitis required a mean operation time of 80 minutes (range: 34–174) and in contrast patients with chronic cholecystitis or no inflammation only 57 minutes (range: 28–159). In addition, we could not indicate a reduction of operation time between the first 50% of operations and the second 50% like Rivas et al. We performed the operation in the first 110 patients in a median time of 61 minutes and the second 110 patients in a median time of 65 minutes. It seems that the learning curve has an important impact but is for an experienced laparoscopic surgeon not as important in large series. But one reason for the more extended time in later operations might be the different view on the indication for single-port surgery after a major experience. The first 30 patients had not undergone previously abdominal surgery and, therefore, the operation easier to perform with a reduced operation time. After increased experience, more severe cases were operated with a higher operation time. Another important point of criticism about single-incision surgery is the conversion rate to multiple ports. Several studies reported about a conversion rate between 0% and 5% and are similar to our results with 2% [13–16, 18, 19]. Only one study of Lee et al. had a high conversion rate of 13.5% because of technical difficulties. Conversion rate to an open procedure was 1% in our study group and is described in the literature with 0% to 2% [13–16, 18, 19]. We had to convert to an open procedure because of an acute bleeding from the cystic artery without an identifiable vessel in the hepatoduodenal ligament. A blind closure of the vessel with 5 mm clips or bipolar thermocoagulation could have injured structures in the ligament. The second patient had an unknown cholecystoduodenal fistula, which could not be closed in laparoscopic technique. Considering these results, the conversion rate in single-incision surgery is even to multiport standard. A view on the complication rates after single-site surgery in the literature shows a percentage between 0% and 5,4% [12–19]. Four studies reported about no complications in their study population [13, 14, 18, 19]. In our study, eight patients (5%) developed postoperative complication, and six of these patients (3.5%) had to undergo reoperation. Except Romanelli et al., who had one case of postoperative hernia, other reports did not mention a reoperation. An analysis of our six patients showed that one of two patients with an incisional hernia had an incidential umbilical hernia and might have used a mesh for optimal wound closure. Two patients developed a wound infection, and a wound debridement had to be performed in both cases. In one patient, the gallbladder was opened for extracting the stone and that might be the reason for infection. If the use of an endobag is more safely for preventing wound infection is questionable. We did not use one endobag in our series and had only an infection rate of 1%. These infections would have healed secondary, but because of a good cosmetic result, we decided to reoperate the patient. In addition, we could identify 31 patients with an incidential umbilical hernia. These hernias could be safely repaired within the standard closure of the fascia using a nonabsorbable suture.

In conclusion, we could demonstrate for the first time that laparoscopic single-incision cholecystectomy as standard procedure is feasible and safe compared to conventional multiport technique. Beside scarless operation, one major advantage in comparison to NOTES is the treatment option for both genders and the use of conventional instruments. Results of long-term followup have to answer the theoretical increased risk of incisional hernia. Therefore, controlled randomized studies are urgently required.
